# Factors Influencing the Achievement of the Critical View of Safety in Laparoscopic Cholecystectomy: A Prospective Observational Study in Yemen

**DOI:** 10.7759/cureus.76222

**Published:** 2024-12-22

**Authors:** Alameen Alnoor, Yasser A Obadiel, Khalil A Saleh, Haitham M Jowah

**Affiliations:** 1 Surgery, Sana’a University, Sana'a City, YEM; 2 Surgery, Al-Kuwait University Hospital, Sana'a City, YEM; 3 Surgery, Sana'a University, Sana'a City, YEM; 4 Surgery, Al-Thawra Modern General Hospital, Sana'a City, YEM; 5 Surgery, Republican Teaching Hospital Authority, Sana'a City, YEM; 6 Surgery, General Military Hospital, Sana'a City, YEM

**Keywords:** bile duct injury, cholecystectomy complications, critical view of safety, difficulty grading, laparoscopic cholecystectomy

## Abstract

Background

The critical view of safety (CVS) is a critical technique to minimize the risk of bile duct injuries (BDIs) during laparoscopic cholecystectomy (LC). This study evaluated the rate of CVS achievement and examined factors influencing its success.

Methods

This prospective study included 97 patients undergoing LC. Data on demographic characteristics, preoperative factors, surgical difficulty, and surgeon experience were collected. CVS achievement was assessed using Strasberg’s criteria, and associated factors were analyzed.

Results

CVS was successfully achieved in 31 of 97 cases (32%), while it was not achieved in 66 cases (68%). Factors significantly associated with failure to achieve CVS included previous abdominal surgery (p = 0.024), prior endoscopic retrograde cholangiopancreatography (ERCP) (p = 0.024), acute cholecystitis (p = 0.024), and higher difficulty grades according to the modified Nassar scale (p < 0.001). Although there was no statistically significant difference in CVS achievement between specialists and residents (p = 0.223), specialists had a higher success rate (37.5%) compared to residents (28%). Achieving CVS was associated with shorter operative times (mean: 60 vs. 70 minutes, p < 0.001) and reduced use of postoperative drains (16.1% vs. 83.9%, p < 0.001). Importantly, no BDIs were observed.

Conclusion

Achieving CVS remains a challenge, particularly in complex cases and patients with prior abdominal interventions or acute inflammation. Enhanced surgical training, meticulous preoperative planning, and the use of adjunctive technologies may improve CVS success rates and contribute to safer outcomes in LC.

## Introduction

Gallstones affect 10%-15% of the adult population, making symptomatic cholelithiasis one of the most prevalent indications for surgical intervention globally [1]. Laparoscopic cholecystectomy (LC), performed approximately 700,000 times annually in the United States alone, has largely supplanted open cholecystectomy. This transition is attributed to the numerous advantages of LC, including superior cosmetic outcomes, reduced length of hospital stay, diminished postoperative pain, and expedited recovery to normal activities [2,3]. Nonetheless, LC is not devoid of risks, including complications such as gallbladder perforation, biliary tract injury, abscess formation, hemorrhage, and pancreatitis [3].

Bile duct injuries (BDIs) are of particular concern, with incidence rates in LC ranging from 0.4% to 1.5% and 0.2% to 0.3% in open cholecystectomy [4]. The critical view of safety (CVS) technique, introduced in 1995, aims to mitigate this risk by precisely identifying the biliary anatomy, notably the hepatocystic triangle [5]. Empirical studies have indicated that accurate identification of CVS significantly reduces the risk of iatrogenic intraoperative complications [6]. However, achieving CVS remains challenging and is achieved in approximately 50% of cases, particularly during the initial learning phase of LC [7,8].

Adherence to CVS during LC in teaching hospitals in Sana’a is a significant issue due to variations in surgeon training levels, limited access to advanced surgical tools, and inconsistent documentation practices within the region. CVS prevents BDIs by ensuring the identification of the cystic duct and artery before clipping and division [6]. BDIs are serious complications that can result in major morbidity, prolonged hospitalization, increased healthcare costs, and legal consequences [9].

Despite the broad recognition of CVS as the standard approach for preventing BDIs, its adoption among surgeons remains variable and often incomplete [10]. There is a paucity of data regarding CVS use and documentation in Yemen, particularly in Sana’a, where LC is performed in numerous teaching hospitals with varying levels of experience and training.

This study focuses on adherence to CVS among surgeons performing LC in teaching hospitals in Sana’a, Yemen. The unique challenges in this setting, including resource limitations, varying levels of surgeon experience, and the reliance on teaching hospitals for surgical training, make achieving CVS particularly complex. By evaluating factors influencing CVS application and documentation and assessing its impact on BDIs and intraoperative complications, this study provides valuable insights into the current practices and challenges in Yemen. Despite involving a relatively small cohort, the findings offer actionable strategies for improving CVS adherence in low-resource and training environments globally, contributing to enhanced LC safety and better patient outcomes in similar contexts.

## Materials and methods

Study design and setting

This prospective observational descriptive study was conducted at four teaching hospitals in Sana’a, Yemen: Al-Thawra Modern General Hospital, Al-Gumhori Teaching Hospital Authority, Al-Kuwait University Hospital, and General Military Hospital. The study period was from November 2023 to January 2024.

Participants

In total, 100 consecutive patients scheduled for elective LC were included in the study. The inclusion criterion was patients aged 18 years and above who consented to participate. Cases involving conversion to open surgery, partial cholecystectomy, or a fundus-first approach were excluded to ensure consistent evaluation of the Strasberg criteria for CVS. While necessary for methodological consistency, this exclusion limits the study’s ability to assess outcomes in high-difficulty cases where achieving CVS may be inherently more challenging.

Data collection

Data were collected prospectively through a combination of medical record review and direct observation of the surgical procedures. The collected data included patient demographics, such as age, sex, American Society of Anesthesiologists (ASA) classification, and comorbidities. Clinical presentation data included symptoms and indications for surgery, including acute cholecystitis and choledocholithiasis. Surgical history was also recorded, noting any previous abdominal surgery and previous endoscopic retrograde cholangiopancreatography (ERCP). Intraoperative details were meticulously documented, including difficulty grading according to the modified Nassar scale [11], surgical experience level (whether the procedure was performed by a resident or specialist), operative time, and intraoperative complications, such as gallbladder perforation, significant bleeding, and bile leakage.

The Nassar scale is a classification system used to assess the difficulty of LC based on anatomical and pathological factors (Table 1).

**Table 1:** Operative difficulty grading: modified Nassar scale

Grade	Description
I	Gallbladder - floppy, non-adherent
	Cystic pedicle - thin and clear
	Adhesions - simple up to the neck/Hartmann pouch
II	Gallbladder - mucocele, packed with stones
	Cystic pedicle - fat-laden
	Adhesions - simple up to the body
III	Gallbladder - deep fossa, acute cholecystitis, contracted, fibrosis, Hartmann’s adherent to common bile duct, impaction
	Cystic pedicle - abnormal anatomy or cystic duct - short, dilated, or obscured
	Adhesions - dense up to fundus; involving hepatic flexure or duodenum
IV	Gallbladder - completely obscured, empyema, gangrene, and mass
	Cystic pedicle - impossible to clarify
	Adhesions - dense, fibrotic, wrapping the gallbladder, duodenum, or hepatic flexure that are difficult to separate
V	Mirizzi syndrome type 2 or higher, cholecystocutaneous, duodenal, or cholecystocolic fistula

Critical view of safety assessment

The CVS during LC was assessed using Strasberg’s criteria [12], a set of guidelines aimed at minimizing the risk of BDI and ensuring the safe identification of critical anatomical structures. These criteria involve three key steps: clearing the hepatobiliary triangle of excess tissue, detaching the lower third of the gallbladder from the liver bed, and separating the cystic duct and artery. Each step was scored as "Not Achieved," "Doubtful," or "Achieved," with a score of five or more indicating successful achievement of CVS (Table 2) [13].

**Table 2:** Strasberg criteria for CVS CVS, critical view of safety; NA, not applicable

Criteria	Scoring		
	0	1	2
Remove excess tissue from the hepatobiliary triangle before disconnecting the duct.	Not Achieved	Doubtful	Achieved
Remove the lower third of the gallbladder bed before duct disconnection.	Not Achieved	N/A	Achieved
Before the duct was dissected, the cystic artery and cystic duct were separated.	Not Achieved	Doubtful	Achieved

This standardized approach reduces the risk of complications, shortens operative times, and improves patient outcomes. Moreover, it serves as an important educational tool for surgeons during training. The independent reviewer in the study underwent specific training to ensure consistency and reliability in applying the criteria throughout the assessment process.

Statistical analysis

The data were analyzed using IBM SPSS Statistics v24.0 (IBM, Armonk, NY, USA). Descriptive statistics, such as frequencies and percentages, were used for categorical variables, whereas means and standard deviations were used for continuous variables. The chi-square test and Fisher’s exact test were employed to assess associations between categorical variables and CVS achievement. The independent t-test was used for continuous variables. A significance level of P < 0.05 was considered statistically significant.

Ethical considerations

The Ethics Committee of Sana’a University, Sana’a, Yemen, approved the study. Informed consent was obtained from all participants, and verbal consent was secured specifically from the surgeons. All procedures were conducted with strict adherence to ethical standards, including those outlined in the Declaration of Helsinki. The confidentiality and anonymity of the participants were maintained throughout the study, ensuring that their personal and medical information was protected. Additionally, the study was designed to minimize potential risks to participants while maximizing the benefits of the research.

## Results

The study included 97 patients, most of whom were female (77.3%), with a median age of 41 years. Most patients were classified as ASA class I (68%). Comorbid conditions, primarily hypertension (21.6%), were present in 31% of the patients (Table 3).

**Table 3:** Demographic and clinical characteristics of the study participants (n=97) ASA, American Society of Anesthesiologists; HTN, hypertension; DM, diabetes mellitus

Characteristic	n (%)
Gender	
Female	75 (77.3%)
Male	22 (22.7%)
Age group	
18-39	40 (41.2%)
40-60	48 (49.5%)
>60	9 (9.3%)
ASA classification	
ASA I	66 (68%)
ASA II	28 (28.9%)
ASA III	3 (3.1%)
Comorbidities	
HTN	21 (21.6%)
DM	6 (6.2%)
Pulmonary diseases	2 (2.1%)
Cardiac diseases	1 (1%)
Hemolytic anemia	1 (1%)

Ultrasonographic findings indicated multiple gallstones in 84.5% of patients, with stones larger than 1 cm detected in 59.8%. Stone impact was observed in 16.5% of the participants (Table 4).

**Table 4:** Preoperative evaluations of the study participants (n=97)

Characteristic	n (%)
Gallstone number	
Single	15 (15.5%)
Multiple	82 (84.5%)
Gallstone size	
<1 cm	39 (40.2%)
>1 cm	58 (59.8%)
Stone impaction	16 (16.5%)
Primary indication for surgery	
Symptomatic gallstones	93 (95.9%)
Choledocholithiasis	2 (2.1%)
Cholangitis	2 (2.1%)

The procedures were performed by residents (58.8%) and specialists (41.2%). The difficulty of the operations was classified as Grade I (59.8%), Grade II (27.8%), Grade III (11.3%), or Grade IV (1.1%), according to the modified Nassar scale. Intraoperative complications included gallbladder perforation (25.7%), significant bleeding (2.1%), and bile leakage (1%) (Table 5).

**Table 5:** Intraoperative findings and complications (n=97)

Characteristic	n (%)
Surgeon experience	
Resident	57 (58.8%)
Specialist	40 (41.2%)
Nassar scale difficulty	
Grade I	58 (59.8%)
Grade II	27 (27.8%)
Grade III	11 (11.3%)
Grade IV	1 (1.1%)
Conversion to open surgery	3 (3%)
Intraoperative complications	
Gallbladder perforation	25 (25.7%)
Significant bleeding	2 (2.1%)
Bile leak	1 (1%)

According to Strasberg’s criteria, individual CVS components were achieved variably, as detailed in Table 6. Overall, CVS was successfully accomplished in 32% of the cases, whereas 68% of the procedures failed to meet the criteria for achieving CVS.

**Table 6:** Achievement of CVS according to the Strasberg criteria CVS, critical view of safety; NA, not applicable

Components of CVS	Not achieved	Inadequate	Achieved	Total
Clearance of the hepatobiliary triangle before duct	1	57	39	97
	1%	58.8%	40.2%	100%
Remove the lower third of the gallbladder bed	33	NA	64	97
	34%		64%	100
Before the duct was dissected, the cystic artery and cystic duct were separated	1	17	79	97
	1%	17.5%	81.4%	100%

The key factors associated with failure to achieve CVS included previous abdominal surgery (P = 0.024), prior ERCP (P = 0.045), acute cholecystitis (P = 0.035), and higher difficulty grades (II-IV) (P < 0.001) (Table 7). Interestingly, our analysis showed no significant difference in CVS achievement between specialists and residents (p = 0.24), although specialists tended to achieve CVS more frequently (37.5% vs. 28%).

**Table 7:** Factors influencing CVS achievement Significant P-values <0.05 *Chi-square test **Fisher’s exact test CVS, critical view of safety; ERCP, endoscopic retrograde cholangiopancreatography

Factor	Achieved, 31 (32%)	Not Achieved, 66 (68%)	P-value
Previous abdominal surgery	1 (12%)	8 (88%)	0.024*
Previous ERCP	0 (0%)	3 (100%)	0.045**
Acute cholecystitis	2 (15%)	11 (85%)	0.035*
Difficulty grade (II-IV)	3 (7.3%)	38 (92.7%)	<0.001*

CVS achievement was associated with various intraoperative and postoperative outcomes. Gallbladder perforation occurred in 12% of cases in which CVS was achieved compared with 88% in cases in which it was not achieved, although the difference was not statistically significant (p = 0.327). The operative time was significantly shorter in patients in whom CVS was achieved, with a mean of 60 min compared to 70 min in the non-achieved group (p < 0.001). Postoperative drain placement was required in 16.1% of cases with successful CVS, compared with 83.9% in the non-achieved group (p < 0.001). The length of hospital stay showed no significant difference between the groups (p = 0.058), although a higher proportion of patients with achieved CVS had shorter hospital stays of one to two days (Table 8).

**Table 8:** Impact of CVS achievement on intraoperative and postoperative outcomes Significant P-values <0.05 *Independent t-test **Chi-square test CVS, critical view of safety

Outcome	Achieved, 31 (32%)	Not achieved, 66 (68%)	P-value
Operative time (minutes)	60 ± 8	70 ± 5	<0.001*
Gallbladder perforation	3 (12%)	22 (88%)	0.327
Drain placement	5 (16.1%)	26 (83.9%)	<0.001**
Hospital stay (days)	1.50 ± 0.18	2.16 ± 0.14	0.058

## Discussion

This study investigated the rate of achieving CVS during LC and identified key factors influencing CVS attainment. The findings revealed that achieving CVS remains a significant challenge in surgical practice, with an overall achievement rate of 32%. This rate is consistent with previously reported CVS success rates, which ranged from 18.18% to 52% in different surgical settings [13-16]. Despite the well-established importance of CVS in preventing BDIs, a critical complication of LC, achieving adequate CVS continues to be difficult in clinical practice, particularly in more complex cases.

A key finding of our study was the significant relationship between the degree of procedural difficulty (graded by the Nassar scale) and the likelihood of achieving CVS. Higher difficulty grades (II-IV) were associated with significantly lower CVS success rates (p < 0.001). This is consistent with previous studies highlighting that more difficult procedures often involve anatomical variations, inflammation, or fibrosis, which complicate the identification of critical structures necessary for CVS [11,13,17,18]. These findings underscore the need for heightened surgical skills and careful preoperative planning when dealing with high-grade cases of difficulty.

Our analysis revealed no statistically significant difference in CVS achievement between specialists and residents (p = 0.327), although specialists achieved CVS more frequently (37.5% vs. 28.1%). This suggests that while surgical experience is important, other factors, such as the complexity of the case, patient-specific challenges, and strict adherence to established protocols, may have a more substantial impact on CVS's success. Additionally, the teaching hospital environment, with its emphasis on training, likely contributes to variability in CVS achievement. Prior studies have demonstrated that targeted training and structured educational programs, such as implementing specialized curricula for CVS, can improve achievement rates significantly, with reported increases from 4% to 42% [19,20]. These findings underscore the necessity of not only accumulating hands-on experience but also providing comprehensive, protocol-based training for surgeons at all levels to enhance their ability to achieve CVS consistently and safely.

The impact of preoperative factors on CVS achievement was also evident in our study, with previous abdominal surgery, acute cholecystitis, and prior ERCP significantly reducing the likelihood of CVS success (p = 0.024, p = 0.035, and p = 0.045, respectively). These findings are consistent with those of previous studies that identified preoperative factors as predictors of CVS achievement during LC. For instance, Gupta et al. [18] reported that factors such as prior surgical history and inflammation significantly impact the ability to achieve CVS, highlighting the importance of thorough preoperative assessment. Similarly, Nijssen et al. [15] reported that complex preoperative conditions, such as previous ERCP, were associated with lower CVS achievement rates, highlighting the need for meticulous preoperative planning. Our findings reinforce the importance of thorough preoperative evaluation and planning, particularly in patients with complex surgical histories or active inflammation. In cases in which achieving CVS is deemed unlikely due to preoperative factors, alternative strategies, such as early conversion to open surgery or the use of intraoperative imaging techniques like indocyanine green cholangiography, may be warranted.

Our study also demonstrated that achieving CVS was associated with several favorable intraoperative and postoperative outcomes. Patients who achieved CVS had significantly shorter operative times (60 minutes vs. 70 minutes, p < 0.001) and a lower need for postoperative drains (16.1% vs. 83.9%, p < 0.001). These findings support the hypothesis that adequate CVS improves surgical efficiency and patient safety. Previous studies have similarly reported that achieving CVS reduces the risk of complications such as BDI, shortens the surgical duration, and may lead to shorter hospital stays [17,21]. Although our study did not find a statistically significant difference in hospital stay duration between the CVS-achieved and non-achieved groups (p = 0.058), the trend toward shorter stays in the CVS-achieved group suggests a potential clinical benefit that may become more apparent in larger studies with extended follow-up.

Despite the challenges in consistently achieving CVS, it is notable that our study did not detect any BDIs. This finding agrees with reports from other studies suggesting that strict adherence to safety protocols can still result in safe outcomes even in cases where CVS is not fully achieved [22,23]. However, the absence of BDIs in this study could also be attributed to the relatively small sample size and the focus on elective surgeries, which may inherently carry a lower risk than emergency procedures.

Our study highlights the ongoing need for enhanced educational programs that focus on CVS techniques. Strategies such as incorporating intraoperative time-outs to ensure meticulous CVS steps and employing adjunctive technologies like near-infrared fluorescence cholangiography can further reduce the risk of BDIs in difficult cases [24-29]. The application of these strategies can serve to further standardize CVS achievement across all surgical skill levels and reduce variability in outcomes, particularly in teaching hospitals where resident involvement is high.

Limitations of this study

This study has several limitations that must be acknowledged. The relatively small sample size and short duration limit the generalizability of the findings, particularly to other populations and healthcare settings. Moreover, the focus on elective surgeries excludes emergency cases, where achieving CVS is often more challenging due to acute inflammation and other complicating factors. Another potential limitation is the Hawthorne effect, where surgeons' behavior may have been influenced by their awareness of being observed, potentially affecting CVS achievement rates [14]. Future studies should aim to address these limitations by including larger and more diverse cohorts, incorporating emergency cases, and extending follow-up periods to provide a more comprehensive understanding of long-term outcomes and complications.

## Conclusions

This study underscores the importance of achieving a CVS during LC to improve patient outcomes and reduce complications. Although the overall rate of CVS achievement remained suboptimal, targeted interventions, including enhanced training and preoperative planning, may help increase success rates. Future research should focus on developing and validating these strategies to further optimize surgical safety and efficacy.
